# Application of a new protocol for providing obstetric care in an outpatient service during the COVID-19 pandemic in a public hospital in Madrid, Spain

**DOI:** 10.3389/fmed.2022.902640

**Published:** 2022-08-03

**Authors:** Maria N. Rayo, Irene Fernández-Buhigas, Emilia Ferrer, María Arrébola, María M. Gil, Belén Santacruz

**Affiliations:** ^1^Department of Obstetrics and Gynecology, Hospital Universitario de Torrejón, Madrid, Spain; ^2^School of Medicine, Universidad Francisco de Vitoria, Madrid, Spain

**Keywords:** pregnancy, protocol, COVID-19 pandemic, obstetric care, SARS-CoV2, outpatient care

## Abstract

**Objective:**

To evaluate the clinical implementation of a preventive COVID-19 protocol regarding re-organization of appointments and documented infections among health workers in an obstetric outpatient service.

**Methods:**

Descriptive analysis of the antenatal care at our obstetric outpatient service and infection rates among health care providers from March 19th to May 22nd, 2020. Appointments were divided into telephone calls or face-to-face examinations. A pre-consultation triage was implemented to identify suspected SARS-CoV2 infected women to reschedule them 14 days later or, if the consultation was non-delayable, to use complete Personal Protective Equipment (PPE). Firstly, the number of face-to-face appointments, telephone appointments, and COVID-19 diagnoses in pregnant women were analyzed. Secondly, the number of obstetricians and nurses diagnosed with SARS-CoV2 infection and their serologic status during universal screening in May 2020 were recorded.

**Results:**

One thousand eight hundred forty-two obstetric appointments were scheduled during this period, including 432 (23.5%) telephone appointments (96.53% according to clinical protocol, 1.62% symptomatic patients advised to stay at home, and 1.85% COVID-19 confirmed cases), and 1,410 (76.5%) face-to-face appointments (9.7% did not attend due to fear of getting the infection, 3.1% were lost-to-follow-up, 0.5% were rescheduled due to COVID-19 symptoms and 86.7% who did attend). Of the 1,223 women attending their hospital appointment, 3.6% screened positive at the triage (72.7% rescheduled and 27.3% seen with PPE). 43 rRT-PCR-SARS-CoV2 tests were performed, and two tested positive. No COVID-19 symptoms were reported among health workers at the outpatient obstetric service, and only one nurse presented immunoglobulin (Ig)G anti-SARS-CoV2.

**Conclusion:**

A prompt implementation of a preventive protocol in a hospital obstetric outpatient service, including triage, hygienic and preventive measurements, and rescheduling pregnancy appointments, reduces the percentage of health workers affected by SARS-CoV2.

## Introduction

On December 31st, 2019, the Authorities of the People's Republic of China reported several pneumonia cases of unknown etiology to the WHO in Wuhan, a city located in the Chinese province of Hubei (1). A week later, they confirmed that it was a new coronavirus named SARS-CoV2, and the disease it causes was named COVID-19 (1). The disease was transmitted similarly to influenza, SARS, or MERS virus, a person-to-person transmission through respiratory drops produced by coughing, sneezing, or speech in close contact. It was also reported that transmission could occur by touching a contaminated surface or any contaminated mucosa (2, 3). Although the contagion mainly occurred through symptomatic people, presymptomatic or very slightly symptomatic patients were also reported infectious (4, 5).

The first case in Spanish territory appeared on January 31st, but it was not until February 24th when the infection with transmission among the population was verified. The first case of local transmission was detected at Hospital Universitario de Torrejón in Madrid, a patient who had been admitted for pneumonia of unknown origin. From that moment, the city of Torrejón de Ardoz, where the hospital is located, became one of the hotspots for spreading infection in the Madrid area. For this reason and to assess the number of people that had been infected during the first peak of the pandemic, the city hall conducted a universal antibody screening undertaken by 75% of the city population (from 1 year old onwards) from May 29th to June 5th, 2020 (6). It was reported that 20.2% of the people presented anti-SARS-CoV2 immunoglobulin (Ig)G (20.3% in women), and 5.1% showed anti-SARS-CoV2 IgM. In contrast, in the subgroup of women of fertile age (15–44 years old), these percentages were 17.1 and 4.5%, respectively.

Since the onset of the disease, one of the major concerns has been the severe risk faced by health care providers, and many different strategies have been implemented to avoid their massive infection. Obstetric outpatient service were high-risk places for exposure since long periods of close contact with the patients are usually required to perform antenatal scans and examinations. Therefore, obstetricians and midwives constituted a particular risk group.

This study aimed to describe the clinical implementation of a preventive protocol in terms of first, re-organization of appointments and hospital visits, and second, the infection rate among health care workers in a hospital obstetric outpatient service during the first peak of the COVID-19 pandemic.

## Materials and methods

### Study design and population

This is a descriptive analysis of the antenatal care provided in the obstetric outpatient service at Hospital Universitario de Torrejón, from March 19th to May 22nd, 2020, during the peak of the first wave of the pandemic in Madrid, Spain. The number of face-to-face appointments, telephone appointments, and COVID-19 diagnoses in pregnant women during that period were analyzed.

Additionally, a universal screening by serological analysis of anti-SARS-CoV2 IgM and IgG was performed on all hospital health workers at the end of May 2020. The number of obstetricians and nurses attending pregnant women at the outpatient service who were diagnosed by rRT-PCR-SARS-CoV2 or presented IgG or IgM anti-SARS-CoV2 at the end of May was recorded.

### Intervention

When the first patient was diagnosed with COVID-19 infection at the hospital on February 24th, 2020, obstetricians assisting pregnant women began to develop a preventive protocol to ensure safety among health care workers and patients. The first protocol version was implemented on March 7th, and the only change applied was related to contact measures. All personnel attending pregnant women in consultations were requested to wear a surgical mask during the entire examination and to take special care in hand hygiene before and after examining each pregnant woman. In addition, it was recommended that a pregnant woman with respiratory symptoms should wear a surgical mask. Since March 14th, the professionals' face mask was changed to an FFP2 (filtering face pieces type 2) if available, and a double pre-consultation triage was implemented. In addition, the government made strict confinement of the population mandatory, so an entire new prenatal care protocol was established to minimize hospital visits and ensure pregnancy care on March 19th. The description of the protocol was as follows:

#### Accompaniment

No companion was allowed in the examination room. The access to the hospital was only limited to the strict necessarily.

#### Triage from the admission department

All pregnant women who required an appointment at the unit received a telephone text message 2–4 days before the visit confirming whether the consultation was face-to-face or by telephone. They were also advised to contact the hospital if they had any symptoms related to the COVID-19 disease. Furthermore, all pregnant women scheduled for a face-to-face appointment were contacted 24 h before their visit to conduct a 7-questions interview concerning COVID-19 (Table 1). If any question was answered positively, the pregnant woman was asked to stay home and wait for the health professional to call her the next day.

**Table 1:** Triage questionnaire.

- Have you had or have a fever or feverish sensation in the last week? - Have you had or have a persistent cough in the last week? - Have you had or have muscle pain in the last week? - Have you had or have general discomfort in the last week? - Does food taste nothing or have you lost the smell? - Are you or have you been positive for COVID-19? If the answer is yes: - Have been passed >5 weeks since the diagnosis? - Do you live or are you in close contact with a patient with COVID-19?

#### Triage upon arrival at consultations

Before entering the outpatient service, a receptionist was performing the same questionnaire (Table 1) again. If all the items in the survey were negative, she was allowed to enter the examination room. The patient was provided with a surgical mask, hand washing facilities, and latex gloves, and she was instructed to keep the security distance (2 empty chairs) in the waiting area.

If any item was positive, she was considered a possible SARS-CoV2 case, and the obstetrician was informed. The procedure was then as follows:

- If the symptoms were severe (fever higher than 37.5 degrees, dyspnea, severe cough, general malaise), the obstetrician referred the patient to the emergency department for clinical assessment without further evaluation, and subsequent management was assessed later.
- If the symptoms were mild, the patient was either rescheduled or assessed with complete PPE if that was a non-delayable appointment (Table 2).

**Table 2:** Rescheduling algorithm for patients screening or testing positive for COVID-19.

**Appointment missed**	**Action**
First-trimester appointment	Rescheduled for US scan 5 weeks later. - Normal ultrasound: cell-free DNA test for aneuploidy screening. - Abnormal^*^ ultrasound: invasive testing.
20–22 weeks anomaly scan	- Fetal anatomy examination by US performed in the first trimester: rescheduled 14 days later. - No fetal anatomy examination by US in the first trimester: anomaly scan rescheduled before 22^+3^ weeks (with PPE).
35–37 growth scan or any other clinically guided scan	Rescheduled 3 weeks later.
Fetal monitoring at 40–41 weeks	- rRT-PCR-SARS-CoV-2 test. - Telephone appointment: ° General well-being, fetal movements, and presence of eventualities inquiry. ° Delivery plan. - Labor induction will be scheduled with all the security measures established if positive rRT-PCR-SARS-COV2.
Invasive test	- Suspected cases: delay the procedure 3 weeks. - Confirmed cases: delay 4 weeks after acute illness. - The procedure cannot be delayed: ° Isolated room ° Health workers with PPE ° The minimum needed personnel
	The risk-benefit was individually assessed.

* Abnormal ultrasound refers to major fetal defects such as holoprosencephaly, omphalocele, megacystis, etc.

US, ultrasound; PPE, personal protection equipment.

#### Follow-up of non-complicated pregnancies

Appointments at the hospital were limited to the strictly necessary. All the appointments were divided into face-to-face and telephone consultations. Those appointments where complementary tests were not required (ultrasound, physical examination, etc.) were carried out by telephone (Table 3). Complementary tests were always performed simultaneously (ultrasound scans, blood tests), and telephone calls were arranged for results if needed.

**Table 3:** Pregnancy monitoring scheme^*^.

**Face-to-face**	**Telephone**
- Symptoms derived ultrasounds. - First-trimester scan and aneuploidy screening (including blood sampling). - 20–22 weeks anomaly scan. - Second-trimester blood sampling (+/- anti-D immunoglobulin administration). - Routine growth scan at 35–37 weeks, Streptococcus B-Agalactiae screening, and blood sampling. - Fetal cardiotocography monitoring at postdates.	- Results from blood analyses or other prenatal tests (including aneuploidy screening). - 30–32 weeks midwife appointment. - 38–39 weeks midwife appointment.

* High-risk cases were managed individually.

#### Ultrasound scans in suspected or confirmed COVID-19 cases

Whenever possible, the obstetric visit was delayed until 14 days after the end of the respiratory symptoms or, at least, the resolution of the active disease (Table 2). If the examination could not be delayed, it was carried out at the end of the session, limiting the number of health workers and the examination time and wearing full PPE (FFP2 or FFP3 masks if available, protected by surgical mask or screen, waterproof gown, double glove, and glasses). The subsequent disinfection of the scanning room was complete, including the ultrasound machine (Table 4).

**Table 4:** Preparation and cleaning of obstetrics room.

Deep cleaning Ultrasound rooms should be cleaned thoroughly (double cleaning) each morning before the arrival of the patients and each evening at the end of the session using CDC-approved cleaners. This cleaning includes: - Computer, keyboard and mouse, printer, door handles, stretcher, chairs, armchairs, ultrasound machines, light switches. - Tensiometer and weight scale. - Fetal monitors.
Ultrasound transducers - The use of ultrasound transducers was limited to only multi-frequency transabdominal ones (one per machine). ° If other transducers were needed (i.e., transvaginal probe): double cleaning should be carried out before storing again. - The transducers in use will be covered with a protector (cover, glove) and will be cleaned with CDC-approved cleaners between patients.
Intermediate cleaning (before the next patient was called into the room) - Hand wash with soap and warm water or with an antimicrobial cleaner for at least 20 s. - Ultrasound transducers and cables disinfection - Patient's bed and chair disinfection - Change disposable gloves (latex-free, polyurethane) - Two pairs of gloves when handling dirty clothes or sheets. Hand wash for at least 20 seconds afterward.

## Results

**Figure 1:**
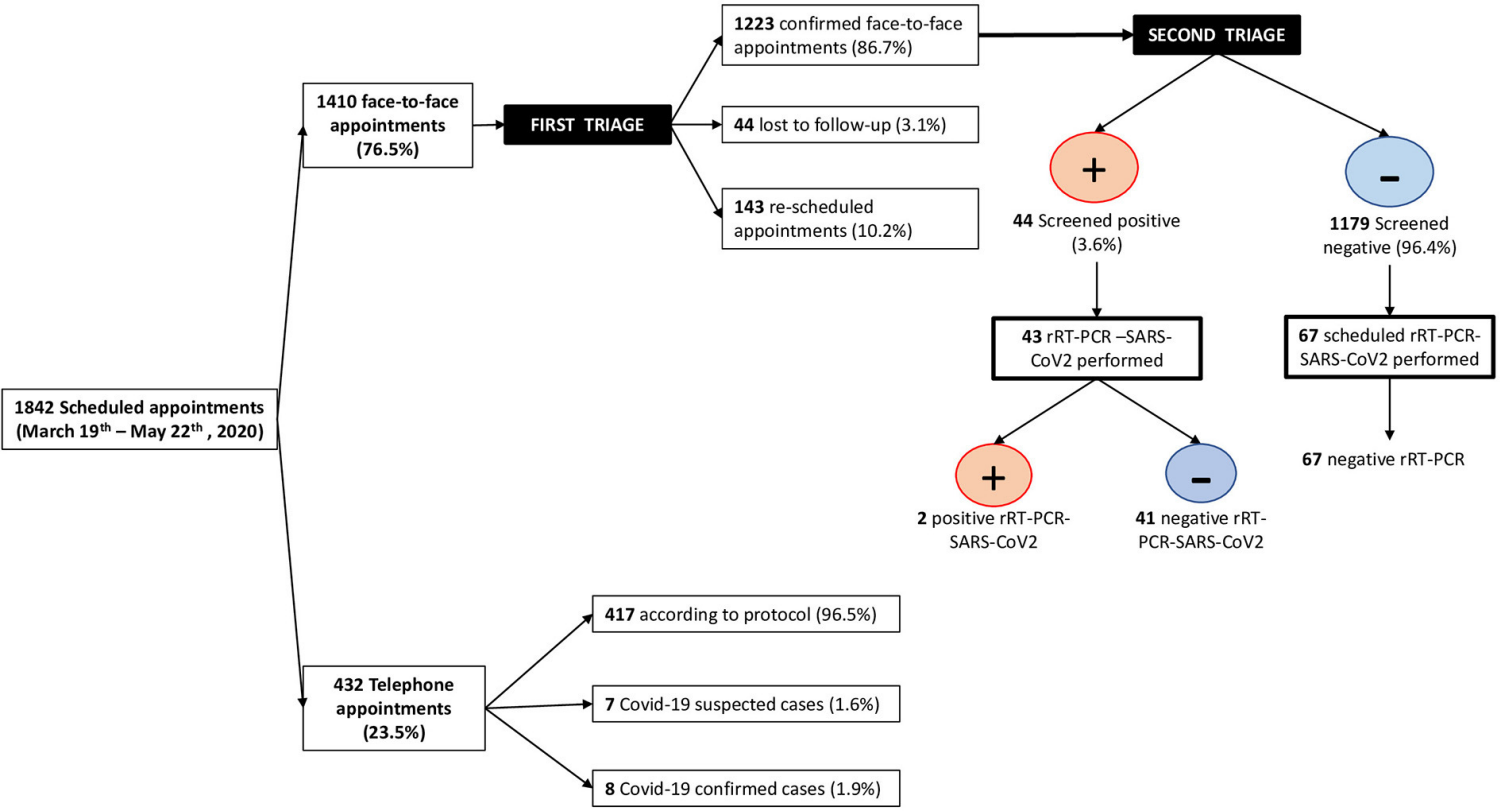
Patients diagram flow.

From March 19th to May 22nd, 2020, 1842 obstetric appointments were scheduled (Figure 1). One thousand four hundred ten (76.5%) were face-to-face appointments and 23.4% were telephone appointments (432 patients). Of the telephone appointments, 417 (96.5%) were telephone calls according to clinical protocol, 7 (1.6%) corresponded to patients that had been advised to stay home as they had a positive item at the first triage, and 8 (1.9%) corresponded to patients who communicated that they were COVID-19 confirmed cases at the first triage. Of the face-to-face scheduled appointments, 137 (9.7%) patients did not attend because they feared getting infected; they were contacted by phone, and a follow-up was arranged. Six (0.5%) women contacted the hospital to report COVID-19 symptoms, and they were rescheduled according to protocol. 44 (3.1%) did not attend and did not answer phone calls, so they were lost to follow up. 1223 (86.7%) attended face-to-face appointments and had a second triage. 44 (3.6%) screened positive, of whom 32 (72.7%) were rescheduled, and 12 (27.3%) were seen on the day.

In 43 (97.7%) of the screened positive pregnant women, an rRT-PCR-SARS-CoV2 test was performed, and 2 of them tested positive. Additionally, 67 screened negative patients at first and second triages, had a planned cesarean section or induction of labor within the study period, and were tested by rRT-PCR-SARS-CoV2 on the day of the appointment according to protocol; none of them tested positive.

In this period, no symptoms of COVID-19 were reported among health care professionals in the outpatient obstetric service, where 12 doctors, 7 nurses/auxiliary nurses, and 3 receptionists worked daily. Anti-SARS-CoV2 IgG and IgM antibodies were only detected in one nurse following universal health care workers' screening, performed at the end of the study period. However, the onset of her symptoms occurred before she started to work in our unit.

## Discussion

### Main findings

The main findings of this study were that, first, a preventive protocol can be quickly implemented following an emergent disease outbreak, but this protocol must be evolving to incorporate new scientific knowledge; second, the implementation of this new protocol in our obstetric outpatient service transformed about 23% of our face-to-face appointments into telephone calls; and third, as a result of the implemented preventive measures, only one health care worker was infected during the first peak of the pandemic.

### Research in context

In Madrid region, official sources pointed out that 11,660 health care professionals, including 3,464 were doctors and 5,789 were nurses or auxiliary nurses, had tested positive by rRT-PCR-SARS-CoV2 (9.772 at hospitals and 1.678 at primary care), and 19 of them had died due to COVID-19 disease by June 15th, 2020 (7).

Two seroprevalence studies carried out at two major referral hospitals in Madrid reported that about 21% and 17% of their health care workers presented anti-SARS-CoV2 antibodies by May and June 2020, respectively (8, 9). The Spanish Ministry of Health sponsored a national seroprevalence study reporting an overall prevalence of antibodies in Spain of 4.6% and in Madrid region in particular of 11.5% in May 2020 (10), confirming that, similarly to other studies, the infection was more prevalent between health care professionals than in the general population (9, 11, 12). However, this was not translated into a higher proportion of infected professionals in our department; our only positive result corresponded to a nurse that was initially working in the emergency department, who presented with symptoms and, after recovery, was transferred to our unit.

Torrejón de Ardoz was a hotspot during the first pandemic's peak and one of the first places where people suffered from COVID-19 disease in Spain. The seroprevalence study carried out in the city showed that about 20% of the population presented anti-SARS-CoV-2 IgG, and about 23% presented any kind of antibody (IgM and IgG) by June 5th, 2020 (6). This study pointed out how severely affected this city was in comparison to the rest of Madrid region. If about 17% of the women of fertile age in the city were exposed to the virus during the study period, we could assume that about 300 (17% of the 1,842 appointments) of the scheduled women at our obstetric service were infected including about 200 (17% of 1,223) who had face-to-face appointments. However, we only identified 44 women as high risk of having COVID-19 in our triage system, and only two had a positive result from the rRT-PCR-SARS-CoV2 for the SARS-CoV2 test. A likely explanation for this lower rate is that first, asymptomatic patients were not tested, and second, most women with symptoms stayed at home. These results highlight the beneficial effect of the early implementation of our preventive protocol. Unfortunately, many other studies have reported delayed actions and much higher rates of infected professionals (4).

### Strengths and limitations

One of the biggest concerns for implementing such a protocol was the decreased quality of care and the subsequent worsening in the perinatal outcomes. However, since that was not the aim of our study, we are not reporting on this. Another limitation relates to the lack of universal testing in pregnant women, which would have been crucial for assessing the usefulness of our protocol in terms of patient safety. However, antibody testing is not a definitive test to determine disease since false positives and negatives may occur.

However, our study presents some significant strengths. First, it is a complete cohort where all scheduled appointments were carefully reviewed, allowing accurate reporting on the protocol consequences in our obstetric service. Second, although not a diagnostic test, all health care professionals at our institution received universal screening for anti-SARS-CoV2 antibodies, which provides invaluable information regarding contagious rate.

### Clinical implications

Since the early implementation of preventive measures was crucial to prevent the spread of the disease, our study has demonstrated the feasibility of rapidly rearranging all appointments within a unit by joining efforts from several professionals (doctors, nurses, and other health workers). Therefore, if a new infection outbreak happened in the future, we would be able to protect health workers and our pregnant population.

## Data availability statement

The raw data supporting the conclusions of this article will be made available by the authors, without undue reservation.

## Ethics statement

Ethical review and approval was not required for the study on human participants in accordance with the local legislation and institutional requirements. Written informed consent from the patients/participants was not required to participate in this study in accordance with the national legislation and the institutional requirements.

## Author contributions

Conceptualization, methodology, and writing—original draft: MR, IF-B, and MG. Data curation and formal analysis: MR and IF-B. Investigation and writing—review and editing: MR, IF-B, EF, MA, BS, and MG. Project administration: MG and BS. Supervision: IF-B, MG, and BS. Validation: MR, IF-B, MG, and BS. All authors contributed to the article and approved the submitted version.

## Conflict of interest

The authors declare that the research was conducted in the absence of any commercial or financial relationships that could be construed as a potential conflict of interest.

## Publisher's note

All claims expressed in this article are solely those of the authors and do not necessarily represent those of their affiliated organizations, or those of the publisher, the editors and the reviewers. Any product that may be evaluated in this article, or claim that may be made by its manufacturer, is not guaranteed or endorsed by the publisher.
